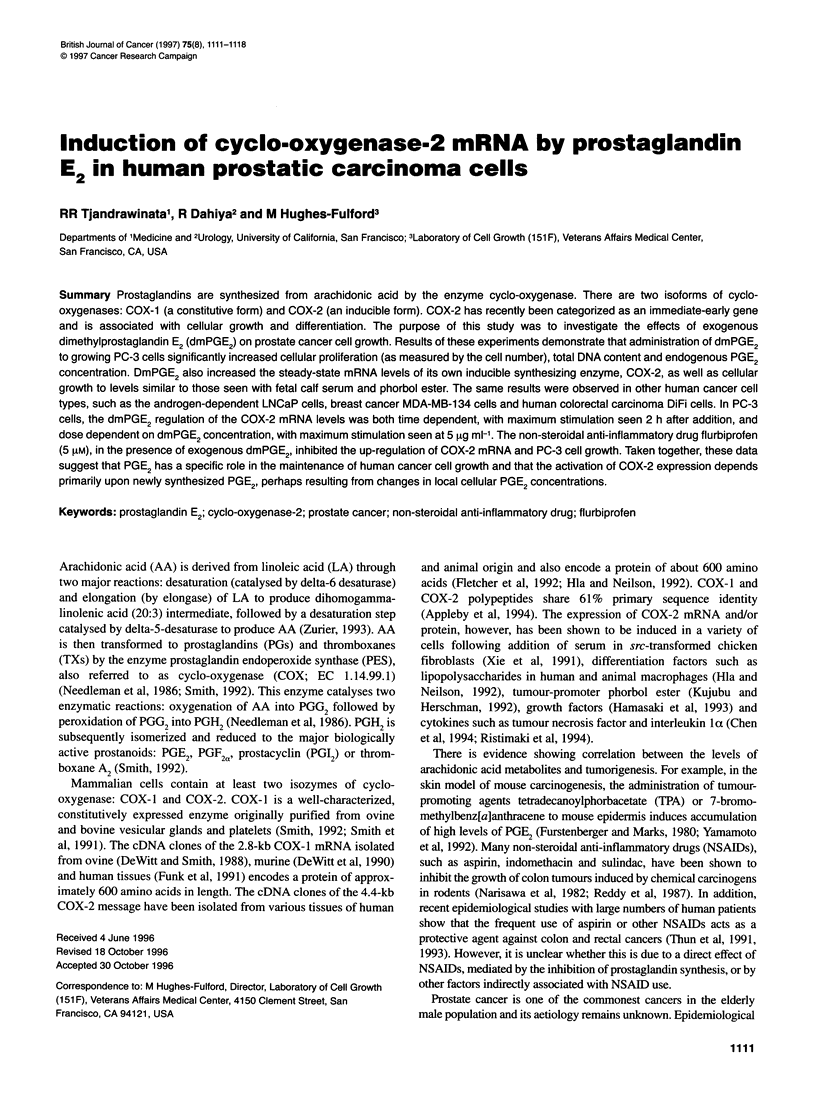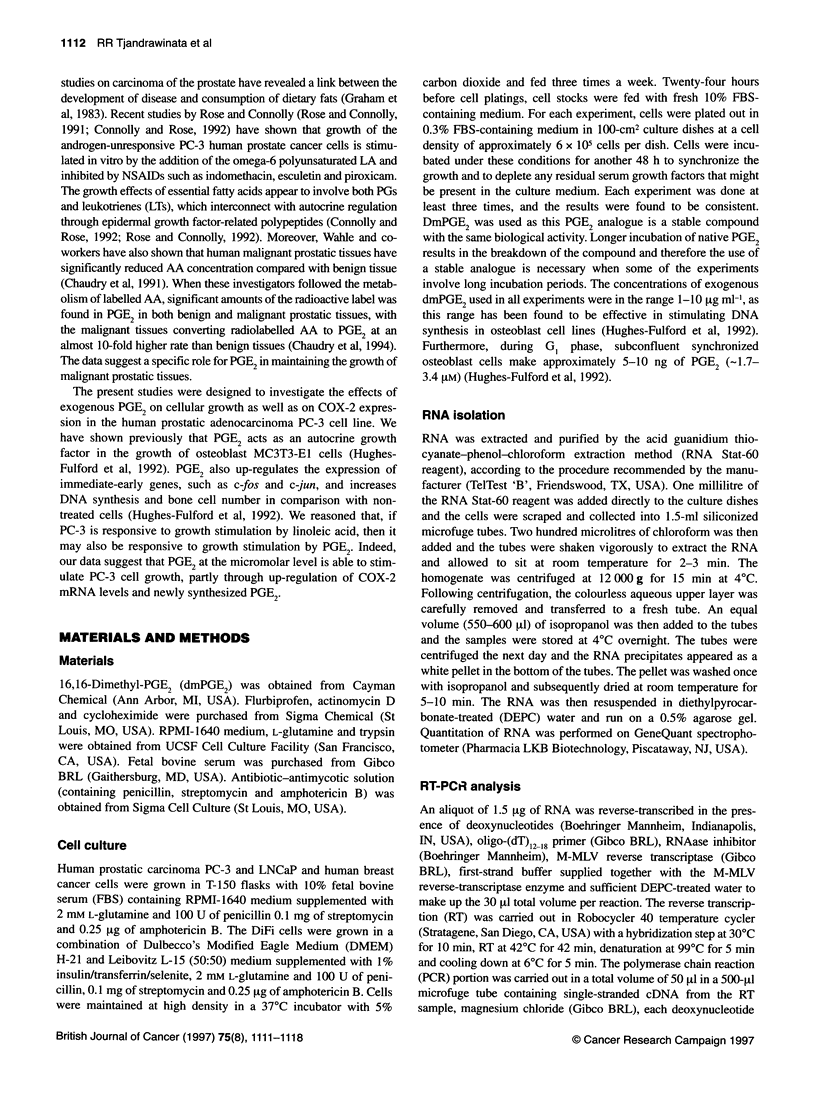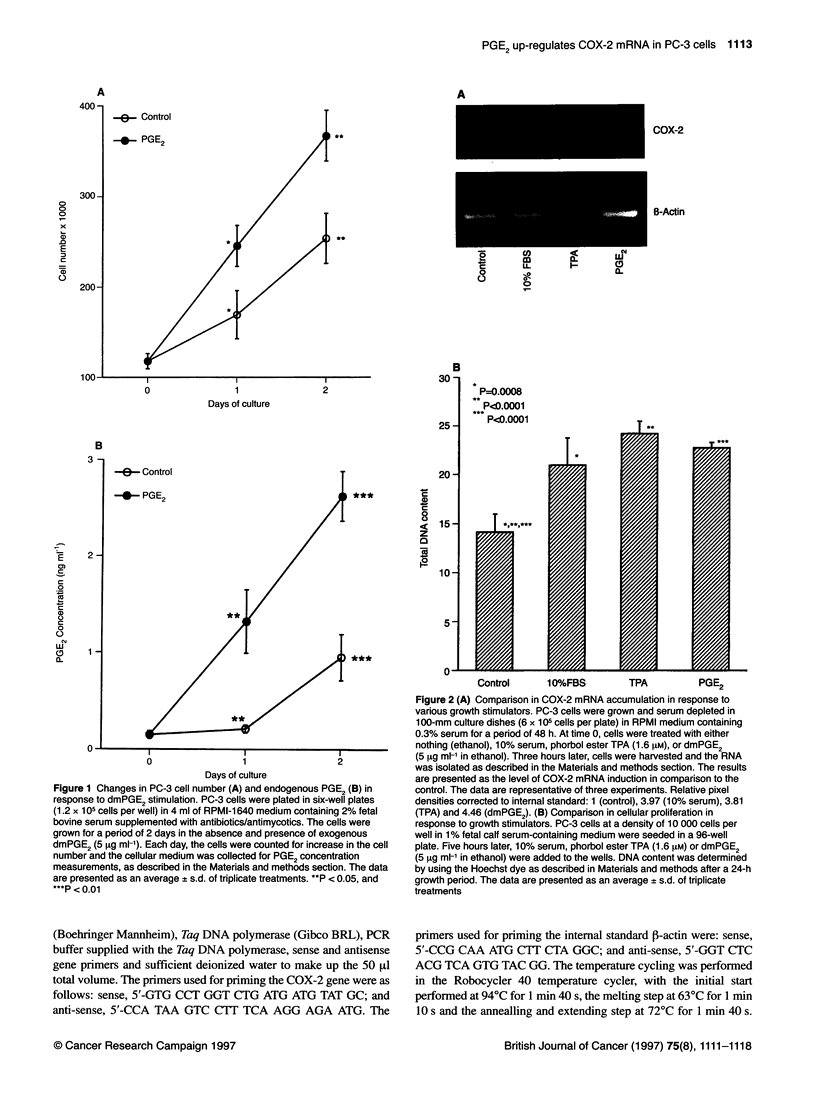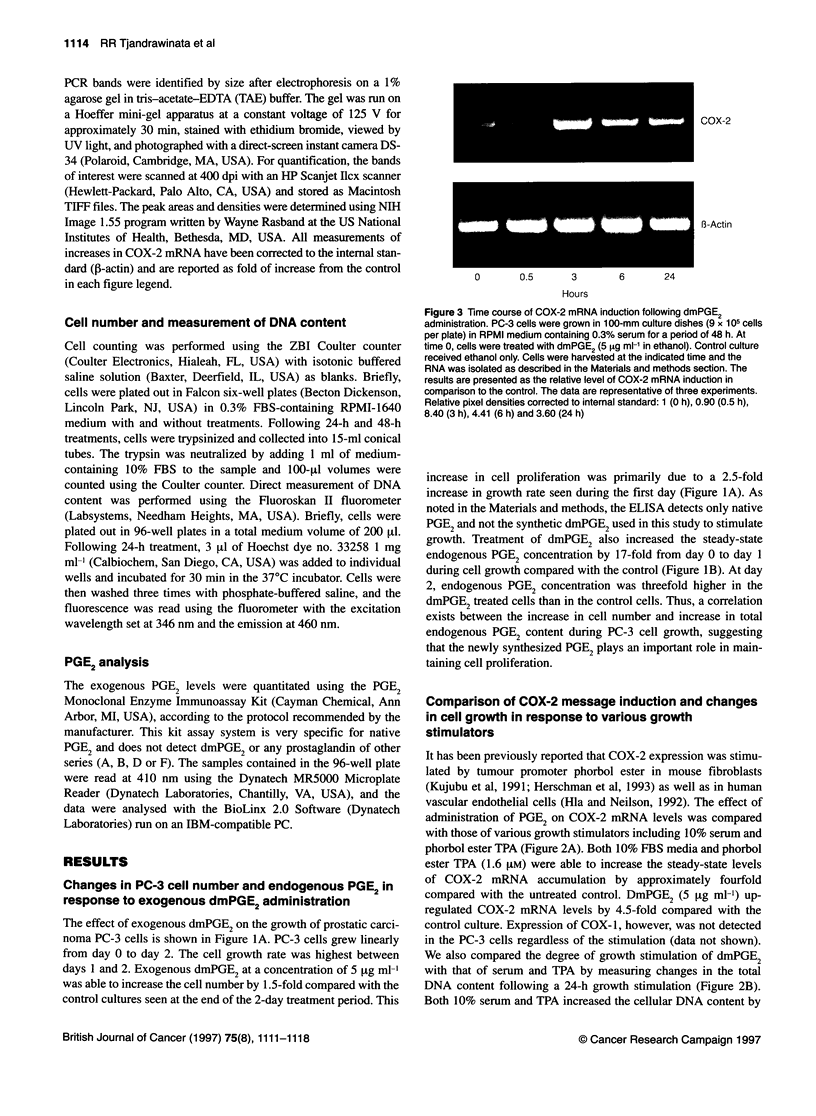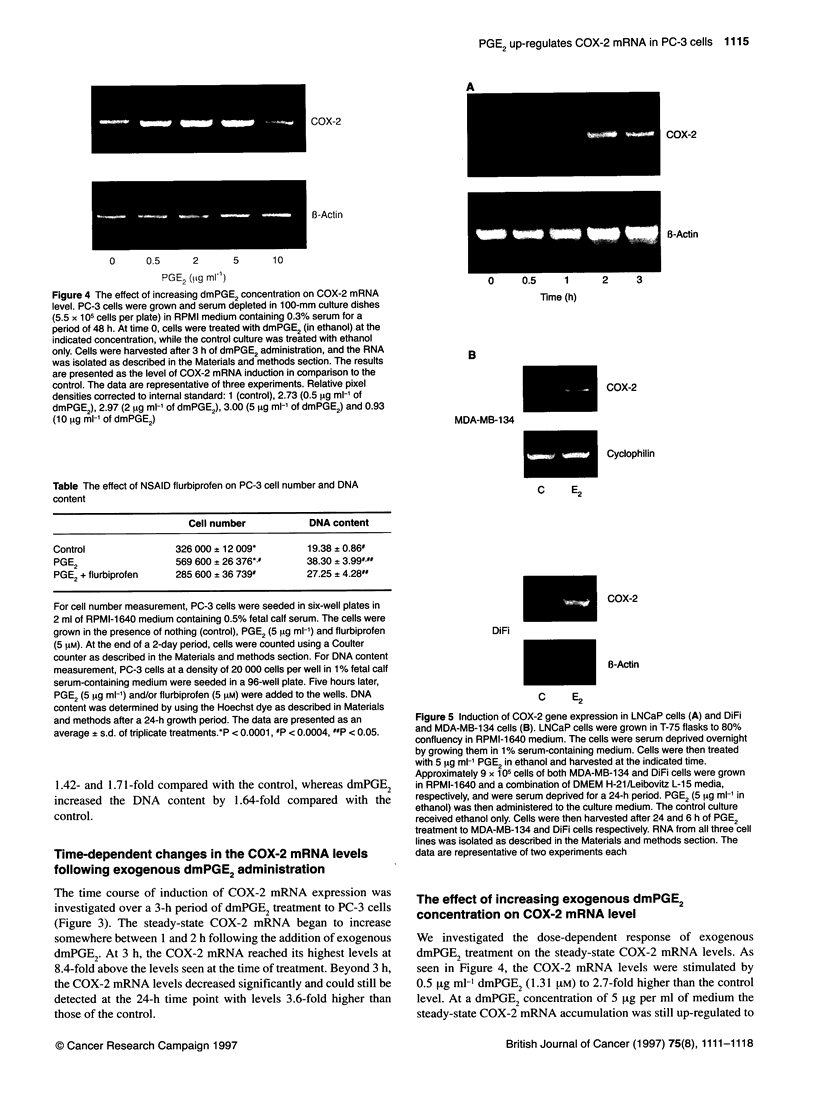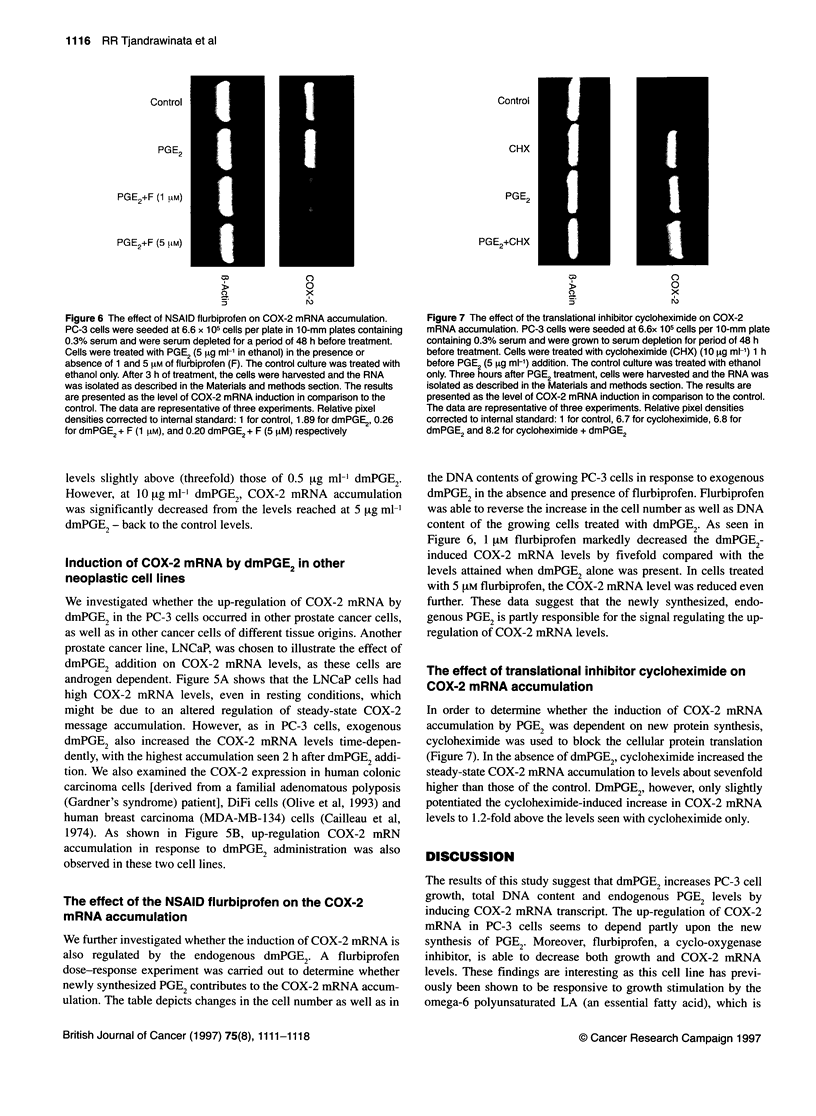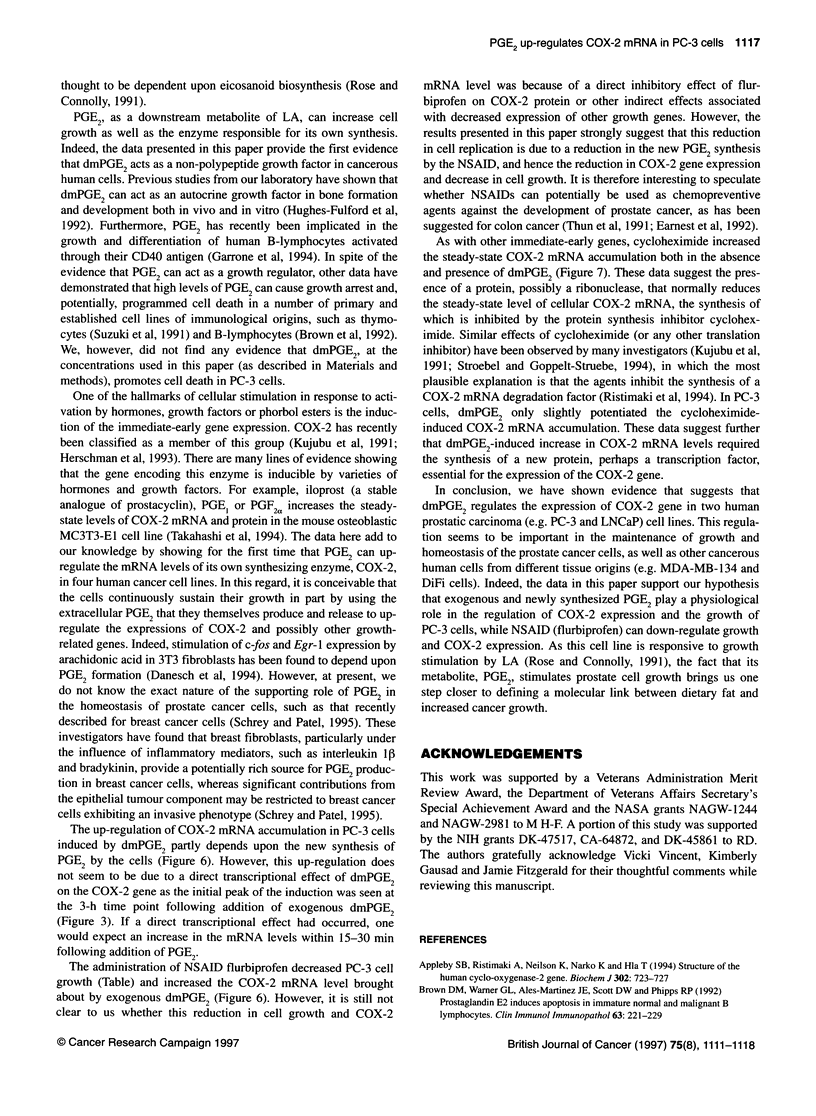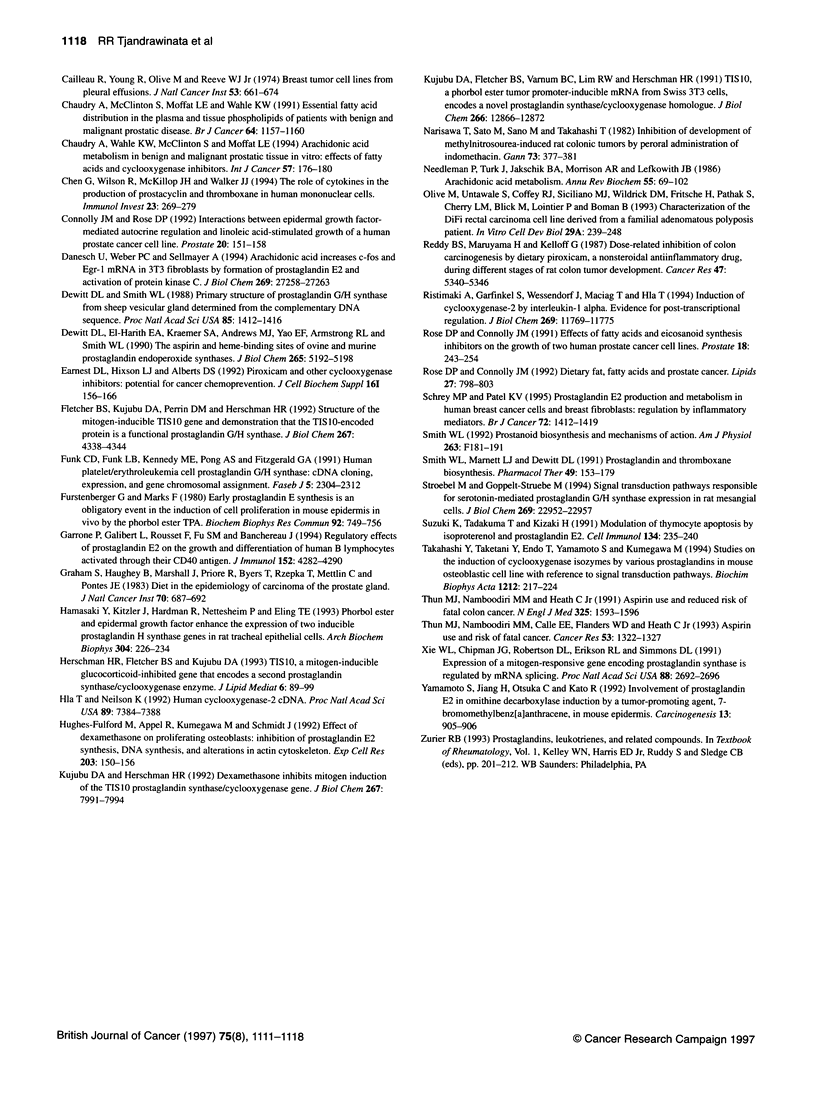# Induction of cyclo-oxygenase-2 mRNA by prostaglandin E2 in human prostatic carcinoma cells.

**DOI:** 10.1038/bjc.1997.192

**Published:** 1997

**Authors:** R. R. Tjandrawinata, R. Dahiya, M. Hughes-Fulford

**Affiliations:** Department of Medicine, University of California, San Francisco, USA.

## Abstract

**Images:**


British Journal of Cancer (1997) 75(8), 1111–1118
© 1997 Cancer Research Campaign

### Induction of cyclo-oxygenase-2 mRNA by prostaglandin $E_2$ in human prostatic carcinoma cells

**RR Tjandrawinata[1], R Dahiya[2] and M Hughes-Fulford[3]**

Departments of [1]Medicine and [2]Urology, University of California, San Francisco; [3]Laboratory of Cell Growth (151F), Veterans Affairs Medical Center, San Francisco, CA, USA

**Summary** Prostaglandins are synthesized from arachidonic acid by the enzyme cyclo-oxygenase. There are two isoforms of cyclo-oxygenases: COX-1 (a constitutive form) and COX-2 (an inducible form). COX-2 has recently been categorized as an immediate-early gene and is associated with cellular growth and differentiation. The purpose of this study was to investigate the effects of exogenous dimethylprostaglandin $E_2$ (dm$PGE_2$) on prostate cancer cell growth. Results of these experiments demonstrate that administration of dm$PGE_2$ to growing PC-3 cells significantly increased cellular proliferation (as measured by the cell number), total DNA content and endogenous $PGE_2$ concentration. Dm$PGE_2$ also increased the steady-state mRNA levels of its own inducible synthesizing enzyme, COX-2, as well as cellular growth to levels similar to those seen with fetal calf serum and phorbol ester. The same results were observed in other human cancer cell types, such as the androgen-dependent LNCaP cells, breast cancer MDA-MB-134 cells and human colorectal carcinoma DiFi cells. In PC-3 cells, the dm$PGE_2$ regulation of the COX-2 mRNA levels was both time dependent, with maximum stimulation seen 2 h after addition, and dose dependent on dm$PGE_2$ concentration, with maximum stimulation seen at 5 $\mu$g ml$^{-1}$. The non-steroidal anti-inflammatory drug flurbiprofen (5 $\mu$M), in the presence of exogenous dm$PGE_2$, inhibited the up-regulation of COX-2 mRNA and PC-3 cell growth. Taken together, these data suggest that $PGE_2$ has a specific role in the maintenance of human cancer cell growth and that the activation of COX-2 expression depends primarily upon newly synthesized $PGE_2$, perhaps resulting from changes in local cellular $PGE_2$ concentrations.

**Keywords:** prostaglandin $E_2$; cyclo-oxygenase-2; prostate cancer; non-steroidal anti-inflammatory drug; flurbiprofen

Arachidonic acid (AA) is derived from linoleic acid (LA) through two major reactions: desaturation (catalysed by delta-6 desaturase) and elongation (by elongase) of LA to produce dihomogamma-linolenic acid (20:3) intermediate, followed by a desaturation step catalysed by delta-5-desaturase to produce AA (Zurier, 1993). AA is then transformed to prostaglandins (PGs) and thromboxanes (TXs) by the enzyme prostaglandin endoperoxide synthase (PES), also referred to as cyclo-oxygenase (COX; EC 1.14.99.1) (Needleman et al, 1986; Smith, 1992). This enzyme catalyses two enzymatic reactions: oxygenation of AA into $PGG_2$ followed by peroxidation of $PGG_2$ into $PGH_2$ (Needleman et al, 1986). $PGH_2$ is subsequently isomerized and reduced to the major biologically active prostanoids: $PGE_2$, $PGF_{2\alpha}$, prostacyclin ($PGI_2$) or thromboxane $A_2$ (Smith, 1992).

Mammalian cells contain at least two isozymes of cyclo-oxygenase: COX-1 and COX-2. COX-1 is a well-characterized, constitutively expressed enzyme originally purified from ovine and bovine vesicular glands and platelets (Smith, 1992; Smith et al, 1991). The cDNA clones of the 2.8-kb COX-1 mRNA isolated from ovine (DeWitt and Smith, 1988), murine (DeWitt et al, 1990) and human tissues (Funk et al, 1991) encodes a protein of approximately 600 amino acids in length. The cDNA clones of the 4.4-kb COX-2 message have been isolated from various tissues of human and animal origin and also encode a protein of about 600 amino acids (Fletcher et al, 1992; Hla and Neilson, 1992). COX-1 and COX-2 polypeptides share 61% primary sequence identity (Appleby et al, 1994). The expression of COX-2 mRNA and/or protein, however, has been shown to be induced in a variety of cells following addition of serum in *src*-transformed chicken fibroblasts (Xie et al, 1991), differentiation factors such as lipopolysaccharides in human and animal macrophages (Hla and Neilson, 1992), tumour-promoter phorbol ester (Kujubu and Herschman, 1992), growth factors (Hamasaki et al, 1993) and cytokines such as tumour necrosis factor and interleukin 1$\alpha$ (Chen et al, 1994; Ristimaki et al, 1994).

There is evidence showing correlation between the levels of arachidonic acid metabolites and tumorigenesis. For example, in the skin model of mouse carcinogenesis, the administration of tumour-promoting agents tetradecanoylphorbacetate (TPA) or 7-bromo-methylbenz[*a*]anthracene to mouse epidermis induces accumulation of high levels of $PGE_2$ (Furstenberger and Marks, 1980; Yamamoto et al, 1992). Many non-steroidal anti-inflammatory drugs (NSAIDs), such as aspirin, indomethacin and sulindac, have been shown to inhibit the growth of colon tumours induced by chemical carcinogens in rodents (Narisawa et al, 1982; Reddy et al, 1987). In addition, recent epidemiological studies with large numbers of human patients show that the frequent use of aspirin or other NSAIDs acts as a protective agent against colon and rectal cancers (Thun et al, 1991, 1993). However, it is unclear whether this is due to a direct effect of NSAIDs, mediated by the inhibition of prostaglandin synthesis, or by other factors indirectly associated with NSAID use.

Prostate cancer is one of the commonest cancers in the elderly male population and its aetiology remains unknown. Epidemiological

Received 4 June 1996
Revised 18 October 1996
Accepted 30 October 1996

Correspondence to: M Hughes-Fulford, Director, Laboratory of Cell Growth (151F), Veterans Affairs Medical Center, 4150 Clement Street, San Francisco, CA 94121, USA

studies on carcinoma of the prostate have revealed a link between the development of disease and consumption of dietary fats (Graham et al, 1983). Recent studies by Rose and Connolly (Rose and Connolly, 1991; Connolly and Rose, 1992) have shown that growth of the androgen-unresponsive PC-3 human prostate cancer cells is stimulated in vitro by the addition of the omega-6 polyunsaturated LA and inhibited by NSAIDs such as indomethacin, esculetin and piroxicam. The growth effects of essential fatty acids appear to involve both PGs and leukotrienes (LTs), which interconnect with autocrine regulation through epidermal growth factor-related polypeptides (Connolly and Rose, 1992; Rose and Connolly, 1992). Moreover, Wahle and coworkers have also shown that human malignant prostatic tissues have significantly reduced AA concentration compared with benign tissue (Chaudry et al, 1991). When these investigators followed the metabolism of labelled AA, significant amounts of the radioactive label was found in $PGE_2$ in both benign and malignant prostatic tissues, with the malignant tissues converting radiolabelled AA to $PGE_2$ at an almost 10-fold higher rate than benign tissues (Chaudry et al, 1994). The data suggest a specific role for $PGE_2$ in maintaining the growth of malignant prostatic tissues.

The present studies were designed to investigate the effects of exogenous $PGE_2$ on cellular growth as well as on COX-2 expression in the human prostatic adenocarcinoma PC-3 cell line. We have shown previously that $PGE_2$ acts as an autocrine growth factor in the growth of osteoblast MC3T3-E1 cells (Hughes-Fulford et al, 1992). $PGE_2$ also up-regulates the expression of immediate-early genes, such as *c-fos* and *c-jun*, and increases DNA synthesis and bone cell number in comparison with non-treated cells (Hughes-Fulford et al, 1992). We reasoned that, if PC-3 is responsive to growth stimulation by linoleic acid, then it may also be responsive to growth stimulation by $PGE_2$. Indeed, our data suggest that $PGE_2$ at the micromolar level is able to stimulate PC-3 cell growth, partly through up-regulation of COX-2 mRNA levels and newly synthesized $PGE_2$.

### MATERIALS AND METHODS

### Materials

16,16-Dimethyl-$PGE_2$ (dm$PGE_2$) was obtained from Cayman Chemical (Ann Arbor, MI, USA). Flurbiprofen, actinomycin D and cycloheximide were purchased from Sigma Chemical (St Louis, MO, USA). RPMI-1640 medium, L-glutamine and trypsin were obtained from UCSF Cell Culture Facility (San Francisco, CA, USA). Fetal bovine serum was purchased from Gibco BRL (Gaithersburg, MD, USA). Antibiotic–antimycotic solution (containing penicillin, streptomycin and amphotericin B) was obtained from Sigma Cell Culture (St Louis, MO, USA).

### Cell culture

Human prostatic carcinoma PC-3 and LNCaP and human breast cancer cells were grown in T-150 flasks with 10% fetal bovine serum (FBS) containing RPMI-1640 medium supplemented with 2 mM L-glutamine and 100 U of penicillin 0.1 mg of streptomycin and 0.25 μg of amphotericin B. The DiFi cells were grown in a combination of Dulbecco's Modified Eagle Medium (DMEM) H-21 and Leibovitz L-15 (50:50) medium supplemented with 1% insulin/transferrin/selenite, 2 mM L-glutamine and 100 U of penicillin, 0.1 mg of streptomycin and 0.25 μg of amphotericin B. Cells were maintained at high density in a 37°C incubator with 5% carbon dioxide and fed three times a week. Twenty-four hours before cell platings, cell stocks were fed with fresh 10% FBS-containing medium. For each experiment, cells were plated out in 0.3% FBS-containing medium in 100-cm² culture dishes at a cell density of approximately $6 \times 10^5$ cells per dish. Cells were incubated under these conditions for another 48 h to synchronize the growth and to deplete any residual serum growth factors that might be present in the culture medium. Each experiment was done at least three times, and the results were found to be consistent. Dm$PGE_2$ was used as this $PGE_2$ analogue is a stable compound with the same biological activity. Longer incubation of native $PGE_2$ results in the breakdown of the compound and therefore the use of a stable analogue is necessary when some of the experiments involve long incubation periods. The concentrations of exogenous dm$PGE_2$ used in all experiments were in the range 1–10 μg $ml^{-1}$, as this range has been found to be effective in stimulating DNA synthesis in osteoblast cell lines (Hughes-Fulford et al, 1992). Furthermore, during $G_1$ phase, subconfluent synchronized osteoblast cells make approximately 5–10 ng of $PGE_2$ (~1.7–3.4 μM) (Hughes-Fulford et al, 1992).

### RNA isolation

RNA was extracted and purified by the acid guanidium thiocyanate–phenol–chloroform extraction method (RNA Stat-60 reagent), according to the procedure recommended by the manufacturer (TelTest 'B', Friendswood, TX, USA). One millilitre of the RNA Stat-60 reagent was added directly to the culture dishes and the cells were scraped and collected into 1.5-ml siliconized microfuge tubes. Two hundred microlitres of chloroform was then added and the tubes were shaken vigorously to extract the RNA and allowed to sit at room temperature for 2–3 min. The homogenate was centrifuged at 12 000 **g** for 15 min at 4°C. Following centrifugation, the colourless aqueous upper layer was carefully removed and transferred to a fresh tube. An equal volume (550–600 μl) of isopropanol was then added to the tubes and the samples were stored at 4°C overnight. The tubes were centrifuged the next day and the RNA precipitates appeared as a white pellet in the bottom of the tubes. The pellet was washed once with isopropanol and subsequently dried at room temperature for 5–10 min. The RNA was then resuspended in diethylpyrocarbonate-treated (DEPC) water and run on a 0.5% agarose gel. Quantitation of RNA was performed on GeneQuant spectrophotometer (Pharmacia LKB Biotechnology, Piscataway, NJ, USA).

### RT-PCR analysis

An aliquot of 1.5 μg of RNA was reverse-transcribed in the presence of deoxynucleotides (Boehringer Mannheim, Indianapolis, IN, USA), oligo-$(dT)_{12-18}$ primer (Gibco BRL), RNAase inhibitor (Boehringer Mannheim), M-MLV reverse transcriptase (Gibco BRL), first-strand buffer supplied together with the M-MLV reverse-transcriptase enzyme and sufficient DEPC-treated water to make up the 30 μl total volume per reaction. The reverse transcription (RT) was carried out in Robocycler 40 temperature cycler (Stratagene, San Diego, CA, USA) with a hybridization step at 30°C for 10 min, RT at 42°C for 42 min, denaturation at 99°C for 5 min and cooling down at 6°C for 5 min. The polymerase chain reaction (PCR) portion was carried out in a total volume of 50 μl in a 500-μl microfuge tube containing single-stranded cDNA from the RT sample, magnesium chloride (Gibco BRL), each deoxynucleotide

© Cancer Research Campaign 1997

Figure 1 Changes in PC-3 cell number (A) and endogenous $PGE_2$ (B) in response to $dmPGE_2$ stimulation. PC-3 cells were plated in six-well plates ($1.2 \times 10^5$ cells per well) in 4 ml of RPMI-1640 medium containing 2% fetal bovine serum supplemented with antibiotics/antimycotics. The cells were grown for a period of 2 days in the absence and presence of exogenous $dmPGE_2$ (5 μg $ml^{-1}$). Each day, the cells were counted for increase in the cell number and the cellular medium was collected for $PGE_2$ concentration measurements, as described in the Materials and methods section. The data are presented as an average ± s.d. of triplicate treatments. **$P < 0.05$, and ***$P < 0.01$

Figure 2 (A) Comparison in COX-2 mRNA accumulation in response to various growth stimulators. PC-3 cells were grown and serum depleted in 100-mm culture dishes ($6 \times 10^5$ cells per plate) in RPMI medium containing 0.3% serum for a period of 48 h. At time 0, cells were treated with either nothing (ethanol), 10% serum, phorbol ester TPA (1.6 μM), or $dmPGE_2$ (5 μg $ml^{-1}$ in ethanol). Three hours later, cells were harvested and the RNA was isolated as described in the Materials and methods section. The results are presented as the level of COX-2 mRNA induction in comparison to the control. The data are representative of three experiments. Relative pixel densities corrected to internal standard: 1 (control), 3.97 (10% serum), 3.81 (TPA) and 4.46 ($dmPGE_2$). (B) Comparison in cellular proliferation in response to growth stimulators. PC-3 cells at a density of 10 000 cells per well in 1% fetal calf serum-containing medium were seeded in a 96-well plate. Five hours later, 10% serum, phorbol ester TPA (1.6 μM) or $dmPGE_2$ (5 μg $ml^{-1}$ in ethanol) were added to the wells. DNA content was determined by using the Hoechst dye as described in Materials and methods after a 24-h growth period. The data are presented as an average ± s.d. of triplicate treatments

(Boehringer Mannheim), *Taq* DNA polymerase (Gibco BRL), PCR buffer supplied with the *Taq* DNA polymerase, sense and antisense gene primers and sufficient deionized water to make up the 50 μl total volume. The primers used for priming the COX-2 gene were as follows: sense, 5′-GTG CCT GGT CTG ATG ATG TAT GC; and anti-sense, 5′-CCA TAA GTC CTT TCA AGG AGA ATG. The primers used for priming the internal standard β-actin were: sense, 5′-CCG CAA ATG CTT CTA GGC; and anti-sense, 5′-GGT CTC ACG TCA GTG TAC GG. The temperature cycling was performed in the Robocycler 40 temperature cycler, with the initial start performed at 94°C for 1 min 40 s, the melting step at 63°C for 1 min 10 s and the annealling and extending step at 72°C for 1 min 40 s.

PCR bands were identified by size after electrophoresis on a 1% agarose gel in tris–acetate–EDTA (TAE) buffer. The gel was run on a Hoeffer mini-gel apparatus at a constant voltage of 125 V for approximately 30 min, stained with ethidium bromide, viewed by UV light, and photographed with a direct-screen instant camera DS-34 (Polaroid, Cambridge, MA, USA). For quantification, the bands of interest were scanned at 400 dpi with an HP Scanjet IIcx scanner (Hewlett-Packard, Palo Alto, CA, USA) and stored as Macintosh TIFF files. The peak areas and densities were determined using NIH Image 1.55 program written by Wayne Rasband at the US National Institutes of Health, Bethesda, MD, USA. All measurements of increases in COX-2 mRNA have been corrected to the internal standard (β-actin) and are reported as fold of increase from the control in each figure legend.

### Cell number and measurement of DNA content

Cell counting was performed using the ZBI Coulter counter (Coulter Electronics, Hialeah, FL, USA) with isotonic buffered saline solution (Baxter, Deerfield, IL, USA) as blanks. Briefly, cells were plated out in Falcon six-well plates (Becton Dickenson, Lincoln Park, NJ, USA) in 0.3% FBS-containing RPMI-1640 medium with and without treatments. Following 24-h and 48-h treatments, cells were trypsinized and collected into 15-ml conical tubes. The trypsin was neutralized by adding 1 ml of medium-containing 10% FBS to the sample and 100-μl volumes were counted using the Coulter counter. Direct measurement of DNA content was performed using the Fluoroskan II fluorometer (Labsystems, Needham Heights, MA, USA). Briefly, cells were plated out in 96-well plates in a total medium volume of 200 μl. Following 24-h treatment, 3 μl of Hoechst dye no. 33258 1 mg ml$^{-1}$ (Calbiochem, San Diego, CA, USA) was added to individual wells and incubated for 30 min in the 37°C incubator. Cells were then washed three times with phosphate-buffered saline, and the fluorescence was read using the fluorometer with the excitation wavelength set at 346 nm and the emission at 460 nm.

### $PGE_2$ analysis

The exogenous $PGE_2$ levels were quantitated using the $PGE_2$ Monoclonal Enzyme Immunoassay Kit (Cayman Chemical, Ann Arbor, MI, USA), according to the protocol recommended by the manufacturer. This kit assay system is very specific for native $PGE_2$ and does not detect dm$PGE_2$ or any prostaglandin of other series (A, B, D or F). The samples contained in the 96-well plate were read at 410 nm using the Dynatech MR5000 Microplate Reader (Dynatech Laboratories, Chantilly, VA, USA), and the data were analysed with the BioLinx 2.0 Software (Dynatech Laboratories) run on an IBM-compatible PC.

### RESULTS

### Changes in PC-3 cell number and endogenous $PGE_2$ in response to exogenous dm$PGE_2$ administration

The effect of exogenous dm$PGE_2$ on the growth of prostatic carcinoma PC-3 cells is shown in Figure 1A. PC-3 cells grew linearly from day 0 to day 2. The cell growth rate was highest between days 1 and 2. Exogenous dm$PGE_2$ at a concentration of 5 μg ml$^{-1}$ was able to increase the cell number by 1.5-fold compared with the control cultures seen at the end of the 2-day treatment period. This increase in cell proliferation was primarily due to a 2.5-fold increase in growth rate seen during the first day (Figure 1A). As noted in the Materials and methods, the ELISA detects only native $PGE_2$ and not the synthetic dm$PGE_2$ used in this study to stimulate growth. Treatment of dm$PGE_2$ also increased the steady-state endogenous $PGE_2$ concentration by 17-fold from day 0 to day 1 during cell growth compared with the control (Figure 1B). At day 2, endogenous $PGE_2$ concentration was threefold higher in the dm$PGE_2$ treated cells than in the control cells. Thus, a correlation exists between the increase in cell number and increase in total endogenous $PGE_2$ content during PC-3 cell growth, suggesting that the newly synthesized $PGE_2$ plays an important role in maintaining cell proliferation.

**Figure 3** Time course of COX-2 mRNA induction following dm$PGE_2$ administration. PC-3 cells were grown in 100-mm culture dishes (9 × $10^5$ cells per plate) in RPMI medium containing 0.3% serum for a period of 48 h. At time 0, cells were treated with dm$PGE_2$ (5 μg ml$^{-1}$ in ethanol). Control culture received ethanol only. Cells were harvested at the indicated time and the RNA was isolated as described in the Materials and methods section. The results are presented as the relative level of COX-2 mRNA induction in comparison to the control. The data are representative of three experiments. Relative pixel densities corrected to internal standard: 1 (0 h), 0.90 (0.5 h), 8.40 (3 h), 4.41 (6 h) and 3.60 (24 h)

### Comparison of COX-2 message induction and changes in cell growth in response to various growth stimulators

It has been previously reported that COX-2 expression was stimulated by tumour promoter phorbol ester in mouse fibroblasts (Kujubu et al, 1991; Herschman et al, 1993) as well as in human vascular endothelial cells (Hla and Neilson, 1992). The effect of administration of $PGE_2$ on COX-2 mRNA levels was compared with those of various growth stimulators including 10% serum and phorbol ester TPA (Figure 2A). Both 10% FBS media and phorbol ester TPA (1.6 μM) were able to increase the steady-state levels of COX-2 mRNA accumulation by approximately fourfold compared with the untreated control. Dm$PGE_2$ (5 μg ml$^{-1}$) up-regulated COX-2 mRNA levels by 4.5-fold compared with the control culture. Expression of COX-1, however, was not detected in the PC-3 cells regardless of the stimulation (data not shown). We also compared the degree of growth stimulation of dm$PGE_2$ with that of serum and TPA by measuring changes in the total DNA content following a 24-h growth stimulation (Figure 2B). Both 10% serum and TPA increased the cellular DNA content by

© Cancer Research Campaign 1997

**Figure 4** The effect of increasing $dmPGE_2$ concentration on COX-2 mRNA level. PC-3 cells were grown and serum depleted in 100-mm culture dishes ($5.5 \times 10^5$ cells per plate) in RPMI medium containing 0.3% serum for a period of 48 h. At time 0, cells were treated with $dmPGE_2$ (in ethanol) at the indicated concentration, while the control culture was treated with ethanol only. Cells were harvested after 3 h of $dmPGE_2$ administration, and the RNA was isolated as described in the Materials and methods section. The results are presented as the level of COX-2 mRNA induction in comparison to the control. The data are representative of three experiments. Relative pixel densities corrected to internal standard: 1 (control), 2.73 (0.5 µg $ml^{-1}$ of $dmPGE_2$), 2.97 (2 µg $ml^{-1}$ of $dmPGE_2$), 3.00 (5 µg $ml^{-1}$ of $dmPGE_2$) and 0.93 (10 µg $ml^{-1}$ of $dmPGE_2$)

**Table** The effect of NSAID flurbiprofen on PC-3 cell number and DNA content

| | Cell number | DNA content |
|---|---|---|
| Control | 326 000 ± 12 009* | 19.38 ± 0.86# |
| $PGE_2$ | 569 600 ± 26 376*,# | 38.30 ± 3.99#,## |
| $PGE_2$ + flurbiprofen | 285 600 ± 36 739# | 27.25 ± 4.28## |

For cell number measurement, PC-3 cells were seeded in six-well plates in 2 ml of RPMI-1640 medium containing 0.5% fetal calf serum. The cells were grown in the presence of nothing (control), $PGE_2$ (5 µg $ml^{-1}$) and flurbiprofen (5 µM). At the end of a 2-day period, cells were counted using a Coulter counter as described in the Materials and methods section. For DNA content measurement, PC-3 cells at a density of 20 000 cells per well in 1% fetal calf serum-containing medium were seeded in a 96-well plate. Five hours later, $PGE_2$ (5 µg $ml^{-1}$) and/or flurbiprofen (5 µM) were added to the wells. DNA content was determined by using the Hoechst dye as described in Materials and methods after a 24-h growth period. The data are presented as an average ± s.d. of triplicate treatments.*$P < 0.0001$, #$P < 0.0004$, ##$P < 0.05$.

1.42- and 1.71-fold compared with the control, whereas $dmPGE_2$ increased the DNA content by 1.64-fold compared with the control.

### Time-dependent changes in the COX-2 mRNA levels following exogenous $dmPGE_2$ administration

The time course of induction of COX-2 mRNA expression was investigated over a 3-h period of $dmPGE_2$ treatment to PC-3 cells (Figure 3). The steady-state COX-2 mRNA began to increase somewhere between 1 and 2 h following the addition of exogenous $dmPGE_2$. At 3 h, the COX-2 mRNA reached its highest levels at 8.4-fold above the levels seen at the time of treatment. Beyond 3 h, the COX-2 mRNA levels decreased significantly and could still be detected at the 24-h time point with levels 3.6-fold higher than those of the control.

**Figure 5** Induction of COX-2 gene expression in LNCaP cells (**A**) and DiFi and MDA-MB-134 cells (**B**). LNCaP cells were grown in T-75 flasks to 80% confluency in RPMI-1640 medium. The cells were serum deprived overnight by growing them in 1% serum-containing medium. Cells were then treated with 5 µg $ml^{-1}$ $PGE_2$ in ethanol and harvested at the indicated time. Approximately $9 \times 10^5$ cells of both MDA-MB-134 and DiFi cells were grown in RPMI-1640 and a combination of DMEM H-21/Leibovitz L-15 media, respectively, and were serum deprived for a 24-h period. $PGE_2$ (5 µg $ml^{-1}$ in ethanol) was then administered to the culture medium. The control culture received ethanol only. Cells were then harvested after 24 and 6 h of $PGE_2$ treatment to MDA-MB-134 and DiFi cells respectively. RNA from all three cell lines was isolated as described in the Materials and methods section. The data are representative of two experiments each

### The effect of increasing exogenous $dmPGE_2$ concentration on COX-2 mRNA level

We investigated the dose-dependent response of exogenous $dmPGE_2$ treatment on the steady-state COX-2 mRNA levels. As seen in Figure 4, the COX-2 mRNA levels were stimulated by 0.5 µg $ml^{-1}$ $dmPGE_2$ (1.31 µM) to 2.7-fold higher than the control level. At a $dmPGE_2$ concentration of 5 µg per ml of medium the steady-state COX-2 mRNA accumulation was still up-regulated to

© Cancer Research Campaign 1997

Figure 6 The effect of NSAID flurbiprofen on COX-2 mRNA accumulation. PC-3 cells were seeded at 6.6 × $10^5$ cells per plate in 10-mm plates containing 0.3% serum and were serum depleted for a period of 48 h before treatment. Cells were treated with $PGE_2$ (5 μg $ml^{-1}$ in ethanol) in the presence or absence of 1 and 5 μM of flurbiprofen (F). The control culture was treated with ethanol only. After 3 h of treatment, the cells were harvested and the RNA was isolated as described in the Materials and methods section. The results are presented as the level of COX-2 mRNA induction in comparison to the control. The data are representative of three experiments. Relative pixel densities corrected to internal standard: 1 for control, 1.89 for $dmPGE_2$, 0.26 for $dmPGE_2$ + F (1 μM), and 0.20 $dmPGE_2$ + F (5 μM) respectively

Figure 7 The effect of the translational inhibitor cycloheximide on COX-2 mRNA accumulation. PC-3 cells were seeded at 6.6× $10^5$ cells per 10-mm plate containing 0.3% serum and were grown to serum depletion for period of 48 h before treatment. Cells were treated with cycloheximide (CHX) (10 μg $ml^{-1}$) 1 h before $PGE_2$ (5 μg $ml^{-1}$) addition. The control culture was treated with ethanol only. Three hours after $PGE_2$ treatment, cells were harvested and the RNA was isolated as described in the Materials and methods section. The results are presented as the level of COX-2 mRNA induction in comparison to the control. The data are representative of three experiments. Relative pixel densities corrected to internal standard: 1 for control, 6.7 for cycloheximide, 6.8 for $dmPGE_2$ and 8.2 for cycloheximide + $dmPGE_2$

levels slightly above (threefold) those of 0.5 μg $ml^{-1}$ $dmPGE_2$. However, at 10 μg $ml^{-1}$ $dmPGE_2$, COX-2 mRNA accumulation was significantly decreased from the levels reached at 5 μg $ml^{-1}$ $dmPGE_2$ – back to the control levels.

### Induction of COX-2 mRNA by $dmPGE_2$ in other neoplastic cell lines

We investigated whether the up-regulation of COX-2 mRNA by $dmPGE_2$ in the PC-3 cells occurred in other prostate cancer cells, as well as in other cancer cells of different tissue origins. Another prostate cancer line, LNCaP, was chosen to illustrate the effect of $dmPGE_2$ addition on COX-2 mRNA levels, as these cells are androgen dependent. Figure 5A shows that the LNCaP cells had high COX-2 mRNA levels, even in resting conditions, which might be due to an altered regulation of steady-state COX-2 message accumulation. However, as in PC-3 cells, exogenous $dmPGE_2$ also increased the COX-2 mRNA levels time-dependently, with the highest accumulation seen 2 h after $dmPGE_2$ addition. We also examined the COX-2 expression in human colonic carcinoma cells [derived from a familial adenomatous polyposis (Gardner's syndrome) patient], DiFi cells (Olive et al, 1993) and human breast carcinoma (MDA-MB-134) cells (Cailleau et al, 1974). As shown in Figure 5B, up-regulation COX-2 mRN accumulation in response to $dmPGE_2$ administration was also observed in these two cell lines.

### The effect of the NSAID flurbiprofen on the COX-2 mRNA accumulation

We further investigated whether the induction of COX-2 mRNA is also regulated by the endogenous $dmPGE_2$. A flurbiprofen dose–response experiment was carried out to determine whether newly synthesized $PGE_2$ contributes to the COX-2 mRNA accumulation. The table depicts changes in the cell number as well as in the DNA contents of growing PC-3 cells in response to exogenous $dmPGE_2$ in the absence and presence of flurbiprofen. Flurbiprofen was able to reverse the increase in the cell number as well as DNA content of the growing cells treated with $dmPGE_2$. As seen in Figure 6, 1 μM flurbiprofen markedly decreased the $dmPGE_2$-induced COX-2 mRNA levels by fivefold compared with the levels attained when $dmPGE_2$ alone was present. In cells treated with 5 μM flurbiprofen, the COX-2 mRNA level was reduced even further. These data suggest that the newly synthesized, endogenous $PGE_2$ is partly responsible for the signal regulating the up-regulation of COX-2 mRNA levels.

### The effect of translational inhibitor cycloheximide on COX-2 mRNA accumulation

In order to determine whether the induction of COX-2 mRNA accumulation by $PGE_2$ was dependent on new protein synthesis, cycloheximide was used to block the cellular protein translation (Figure 7). In the absence of $dmPGE_2$, cycloheximide increased the steady-state COX-2 mRNA accumulation to levels about sevenfold higher than those of the control. $DmPGE_2$, however, only slightly potentiated the cycloheximide-induced increase in COX-2 mRNA levels to 1.2-fold above the levels seen with cycloheximide only.

### DISCUSSION

The results of this study suggest that $dmPGE_2$ increases PC-3 cell growth, total DNA content and endogenous $PGE_2$ levels by inducing COX-2 mRNA transcript. The up-regulation of COX-2 mRNA in PC-3 cells seems to depend partly upon the new synthesis of $PGE_2$. Moreover, flurbiprofen, a cyclo-oxygenase inhibitor, is able to decrease both growth and COX-2 mRNA levels. These findings are interesting as this cell line has previously been shown to be responsive to growth stimulation by the omega-6 polyunsaturated LA (an essential fatty acid), which is

© Cancer Research Campaign 1997

thought to be dependent upon eicosanoid biosynthesis (Rose and Connolly, 1991).

$PGE_2$, as a downstream metabolite of LA, can increase cell growth as well as the enzyme responsible for its own synthesis. Indeed, the data presented in this paper provide the first evidence that $dmPGE_2$ acts as a non-polypeptide growth factor in cancerous human cells. Previous studies from our laboratory have shown that $dmPGE_2$ can act as an autocrine growth factor in bone formation and development both in vivo and in vitro (Hughes-Fulford et al, 1992). Furthermore, $PGE_2$ has recently been implicated in the growth and differentiation of human B-lymphocytes activated through their CD40 antigen (Garrone et al, 1994). In spite of the evidence that $PGE_2$ can act as a growth regulator, other data have demonstrated that high levels of $PGE_2$ can cause growth arrest and, potentially, programmed cell death in a number of primary and established cell lines of immunological origins, such as thymocytes (Suzuki et al, 1991) and B-lymphocytes (Brown et al, 1992). We, however, did not find any evidence that $dmPGE_2$, at the concentrations used in this paper (as described in Materials and methods), promotes cell death in PC-3 cells.

One of the hallmarks of cellular stimulation in response to activation by hormones, growth factors or phorbol esters is the induction of the immediate-early gene expression. COX-2 has recently been classified as a member of this group (Kujubu et al, 1991; Herschman et al, 1993). There are many lines of evidence showing that the gene encoding this enzyme is inducible by varieties of hormones and growth factors. For example, iloprost (a stable analogue of prostacyclin), $PGE_1$ or $PGF_{2\alpha}$ increases the steady-state levels of COX-2 mRNA and protein in the mouse osteoblastic MC3T3-E1 cell line (Takahashi et al, 1994). The data here add to our knowledge by showing for the first time that $PGE_2$ can up-regulate the mRNA levels of its own synthesizing enzyme, COX-2, in four human cancer cell lines. In this regard, it is conceivable that the cells continuously sustain their growth in part by using the extracellular $PGE_2$ that they themselves produce and release to up-regulate the expressions of COX-2 and possibly other growth-related genes. Indeed, stimulation of *c-fos* and *Egr-1* expression by arachidonic acid in 3T3 fibroblasts has been found to depend upon $PGE_2$ formation (Danesch et al, 1994). However, at present, we do not know the exact nature of the supporting role of $PGE_2$ in the homeostasis of prostate cancer cells, such as that recently described for breast cancer cells (Schrey and Patel, 1995). These investigators have found that breast fibroblasts, particularly under the influence of inflammatory mediators, such as interleukin 1β and bradykinin, provide a potentially rich source for $PGE_2$ production in breast cancer cells, whereas significant contributions from the epithelial tumour component may be restricted to breast cancer cells exhibiting an invasive phenotype (Schrey and Patel, 1995).

The up-regulation of COX-2 mRNA accumulation in PC-3 cells induced by $dmPGE_2$ partly depends upon the new synthesis of $PGE_2$ by the cells (Figure 6). However, this up-regulation does not seem to be due to a direct transcriptional effect of $dmPGE_2$ on the COX-2 gene as the initial peak of the induction was seen at the 3-h time point following addition of exogenous $dmPGE_2$ (Figure 3). If a direct transcriptional effect had occurred, one would expect an increase in the mRNA levels within 15–30 min following addition of $PGE_2$.

The administration of NSAID flurbiprofen decreased PC-3 cell growth (Table) and increased the COX-2 mRNA level brought about by exogenous $dmPGE_2$ (Figure 6). However, it is still not clear to us whether this reduction in cell growth and COX-2 mRNA level was because of a direct inhibitory effect of flurbiprofen on COX-2 protein or other indirect effects associated with decreased expression of other growth genes. However, the results presented in this paper strongly suggest that this reduction in cell replication is due to a reduction in the new $PGE_2$ synthesis by the NSAID, and hence the reduction in COX-2 gene expression and decrease in cell growth. It is therefore interesting to speculate whether NSAIDs can potentially be used as chemopreventive agents against the development of prostate cancer, as has been suggested for colon cancer (Thun et al, 1991; Earnest et al, 1992).

As with other immediate-early genes, cycloheximide increased the steady-state COX-2 mRNA accumulation both in the absence and presence of $dmPGE_2$ (Figure 7). These data suggest the presence of a protein, possibly a ribonuclease, that normally reduces the steady-state level of cellular COX-2 mRNA, the synthesis of which is inhibited by the protein synthesis inhibitor cycloheximide. Similar effects of cycloheximide (or any other translation inhibitor) have been observed by many investigators (Kujubu et al, 1991; Stroebel and Goppelt-Struebe, 1994), in which the most plausible explanation is that the agents inhibit the synthesis of a COX-2 mRNA degradation factor (Ristimaki et al, 1994). In PC-3 cells, $dmPGE_2$ only slightly potentiated the cycloheximide-induced COX-2 mRNA accumulation. These data suggest further that $dmPGE_2$-induced increase in COX-2 mRNA levels required the synthesis of a new protein, perhaps a transcription factor, essential for the expression of the COX-2 gene.

In conclusion, we have shown evidence that suggests that $dmPGE_2$ regulates the expression of COX-2 gene in two human prostatic carcinoma (e.g. PC-3 and LNCaP) cell lines. This regulation seems to be important in the maintenance of growth and homeostasis of the prostate cancer cells, as well as other cancerous human cells from different tissue origins (e.g. MDA-MB-134 and DiFi cells). Indeed, the data in this paper support our hypothesis that exogenous and newly synthesized $PGE_2$ play a physiological role in the regulation of COX-2 expression and the growth of PC-3 cells, while NSAID (flurbiprofen) can down-regulate growth and COX-2 expression. As this cell line is responsive to growth stimulation by LA (Rose and Connolly, 1991), the fact that its metabolite, $PGE_2$, stimulates prostate cell growth brings us one step closer to defining a molecular link between dietary fat and increased cancer growth.

### ACKNOWLEDGEMENTS

This work was supported by a Veterans Administration Merit Review Award, the Department of Veterans Affairs Secretary's Special Achievement Award and the NASA grants NAGW-1244 and NAGW-2981 to M H-F. A portion of this study was supported by the NIH grants DK-47517, CA-64872, and DK-45861 to RD. The authors gratefully acknowledge Vicki Vincent, Kimberly Gausad and Jamie Fitzgerald for their thoughtful comments while reviewing this manuscript.

### REFERENCES

Appleby SB, Ristimaki A, Neilson K, Narko K and Hla T (1994) Structure of the human cyclo-oxygenase-2 gene. *Biochem J* **302**: 723–727

Brown DM, Warner GL, Ales-Martinez JE, Scott DW and Phipps RP (1992) Prostaglandin E2 induces apoptosis in immature normal and malignant B lymphocytes. *Clin Immunol Immunopathol* **63**: 221–229

© Cancer Research Campaign 1997 

Cailleau R, Young R, Olive M and Reeve WJ Jr (1974) Breast tumor cell lines from pleural effusions. *J Natl Cancer Inst* **53**: 661–674

Chaudry A, McClinton S, Moffat LE and Wahle KW (1991) Essential fatty acid distribution in the plasma and tissue phospholipids of patients with benign and malignant prostatic disease. *Br J Cancer* **64**: 1157–1160

Chaudry A, Wahle KW, McClinton S and Moffat LE (1994) Arachidonic acid metabolism in benign and malignant prostatic tissue in vitro: effects of fatty acids and cyclooxygenase inhibitors. *Int J Cancer* **57**: 176–180

Chen G, Wilson R, McKillop JH and Walker JJ (1994) The role of cytokines in the production of prostacyclin and thromboxane in human mononuclear cells. *Immunol Invest* **23**: 269–279

Connolly JM and Rose DP (1992) Interactions between epidermal growth factor-mediated autocrine regulation and linoleic acid-stimulated growth of a human prostate cancer cell line. *Prostate* **20**: 151–158

Danesch U, Weber PC and Sellmayer A (1994) Arachidonic acid increases c-fos and Egr-1 mRNA in 3T3 fibroblasts by formation of prostaglandin E2 and activation of protein kinase C. *J Biol Chem* **269**: 27258–27263

Dewitt DL and Smith WL (1988) Primary structure of prostaglandin G/H synthase from sheep vesicular gland determined from the complementary DNA sequence. *Proc Natl Acad Sci USA* **85**: 1412–1416

Dewitt DL, El-Harith EA, Kraemer SA, Andrews MJ, Yao EF, Armstrong RL and Smith WL (1990) The aspirin and heme-binding sites of ovine and murine prostaglandin endoperoxide synthases. *J Biol Chem* **265**: 5192–5198

Earnest DL, Hixson LJ and Alberts DS (1992) Piroxicam and other cyclooxygenase inhibitors: potential for cancer chemoprevention. *J Cell Biochem Suppl* **16I** 156–166

Fletcher BS, Kujubu DA, Perrin DM and Herschman HR (1992) Structure of the mitogen-inducible TIS10 gene and demonstration that the TIS10-encoded protein is a functional prostaglandin G/H synthase. *J Biol Chem* **267**: 4338–4344

Funk CD, Funk LB, Kennedy ME, Pong AS and Fitzgerald GA (1991) Human platelet/erythroleukemia cell prostaglandin G/H synthase: cDNA cloning, expression, and gene chromosomal assignment. *Faseb J* **5**: 2304–2312

Furstenberger G and Marks F (1980) Early prostaglandin E synthesis is an obligatory event in the induction of cell proliferation in mouse epidermis in vivo by the phorbol ester TPA. *Biochem Biophys Res Commun* **92**: 749–756

Garrone P, Galibert L, Rousset F, Fu SM and Banchereau J (1994) Regulatory effects of prostaglandin E2 on the growth and differentiation of human B lymphocytes activated through their CD40 antigen. *J Immunol* **152**: 4282–4290

Graham S, Haughey B, Marshall J, Priore R, Byers T, Rzepka T, Mettlin C and Pontes JE (1983) Diet in the epidemiology of carcinoma of the prostate gland. *J Natl Cancer Inst* **70**: 687–692

Hamasaki Y, Kitzler J, Hardman R, Nettesheim P and Eling TE (1993) Phorbol ester and epidermal growth factor enhance the expression of two inducible prostaglandin H synthase genes in rat tracheal epithelial cells. *Arch Biochem Biophys* **304**: 226–234

Herschman HR, Fletcher BS and Kujubu DA (1993) TIS10, a mitogen-inducible glucocorticoid-inhibited gene that encodes a second prostaglandin synthase/cyclooxygenase enzyme. *J Lipid Mediat* **6**: 89–99

Hla T and Neilson K (1992) Human cyclooxygenase-2 cDNA. *Proc Natl Acad Sci USA* **89**: 7384–7388

Hughes-Fulford M, Appel R, Kumegawa M and Schmidt J (1992) Effect of dexamethasone on proliferating osteoblasts: inhibition of prostaglandin E2 synthesis, DNA synthesis, and alterations in actin cytoskeleton. *Exp Cell Res* **203**: 150–156

Kujubu DA and Herschman HR (1992) Dexamethasone inhibits mitogen induction of the TIS10 prostaglandin synthase/cyclooxygenase gene. *J Biol Chem* **267**: 7991–7994

Kujubu DA, Fletcher BS, Varnum BC, Lim RW and Herschman HR (1991) TIS10, a phorbol ester tumor promoter-inducible mRNA from Swiss 3T3 cells, encodes a novel prostaglandin synthase/cyclooxygenase homologue. *J Biol Chem* **266**: 12866–12872

Narisawa T, Sato M, Sano M and Takahashi T (1982) Inhibition of development of methylnitrosourea-induced rat colonic tumors by peroral administration of indomethacin. *Gann* **73**: 377–381

Needleman P, Turk J, Jakschik BA, Morrison AR and Lefkowith JB (1986) Arachidonic acid metabolism. *Annu Rev Biochem* **55**: 69–102

Olive M, Untawale S, Coffey RJ, Siciliano MJ, Wildrick DM, Fritsche H, Pathak S, Cherry LM, Blick M, Lointier P and Boman B (1993) Characterization of the DiFi rectal carcinoma cell line derived from a familial adenomatous polyposis patient. *In Vitro Cell Dev Biol* **29A**: 239–248

Reddy BS, Maruyama H and Kelloff G (1987) Dose-related inhibition of colon carcinogenesis by dietary piroxicam, a nonsteroidal antiinflammatory drug, during different stages of rat colon tumor development. *Cancer Res* **47**: 5340–5346

Ristimaki A, Garfinkel S, Wessendorf J, Maciag T and Hla T (1994) Induction of cyclooxygenase-2 by interleukin-1 alpha. Evidence for post-transcriptional regulation. *J Biol Chem* **269**: 11769–11775

Rose DP and Connolly JM (1991) Effects of fatty acids and eicosanoid synthesis inhibitors on the growth of two human prostate cancer cell lines. *Prostate* **18**: 243–254

Rose DP and Connolly JM (1992) Dietary fat, fatty acids and prostate cancer. *Lipids* **27**: 798–803

Schrey MP and Patel KV (1995) Prostaglandin E2 production and metabolism in human breast cancer cells and breast fibroblasts: regulation by inflammatory mediators. *Br J Cancer* **72**: 1412–1419

Smith WL (1992) Prostanoid biosynthesis and mechanisms of action. *Am J Physiol* **263**: F181–191

Smith WL, Marnett LJ and Dewitt DL (1991) Prostaglandin and thromboxane biosynthesis. *Pharmacol Ther* **49**: 153–179

Stroebel M and Goppelt-Struebe M (1994) Signal transduction pathways responsible for serotonin-mediated prostaglandin G/H synthase expression in rat mesangial cells. *J Biol Chem* **269**: 22952–22957

Suzuki K, Tadakuma T and Kizaki H (1991) Modulation of thymocyte apoptosis by isoproterenol and prostaglandin E2. *Cell Immunol* **134**: 235–240

Takahashi Y, Taketani Y, Endo T, Yamamoto S and Kumegawa M (1994) Studies on the induction of cyclooxygenase isozymes by various prostaglandins in mouse osteoblastic cell line with reference to signal transduction pathways. *Biochim Biophys Acta* **1212**: 217–224

Thun MJ, Namboodiri MM and Heath C Jr (1991) Aspirin use and reduced risk of fatal colon cancer. *N Engl J Med* **325**: 1593–1596

Thun MJ, Namboodiri MM, Calle EE, Flanders WD and Heath C Jr (1993) Aspirin use and risk of fatal cancer. *Cancer Res* **53**: 1322–1327

Xie WL, Chipman JG, Robertson DL, Erikson RL and Simmons DL (1991) Expression of a mitogen-responsive gene encoding prostaglandin synthase is regulated by mRNA splicing. *Proc Natl Acad Sci USA* **88**: 2692–2696

Yamamoto S, Jiang H, Otsuka C and Kato R (1992) Involvement of prostaglandin E2 in omithine decarboxylase induction by a tumor-promoting agent, 7-bromomethylbenz[a]anthracene, in mouse epidermis. *Carcinogenesis* **13**: 905–906

Zurier RB (1993) Prostaglandins, leukotrienes, and related compounds. In *Textbook of Rheumatology*, Vol. 1, Kelley WN, Harris ED Jr, Ruddy S and Sledge CB (eds), pp. 201–212. WB Saunders: Philadelphia, PA

© Cancer Research Campaign 1997